# Challenges of intracellular visualization using virtual and augmented reality

**DOI:** 10.3389/fbinf.2022.997082

**Published:** 2022-09-13

**Authors:** Cesar Augusto Valades-Cruz, Ludovic Leconte, Gwendal Fouche, Thomas Blanc, Nathan Van Hille, Kevin Fournier, Tao Laurent, Benjamin Gallean, Francois Deslandes, Bassam Hajj, Emmanuel Faure, Ferran Argelaguet, Alain Trubuil, Tobias Isenberg, Jean-Baptiste Masson, Jean Salamero, Charles Kervrann

**Affiliations:** ^1^ SERPICO Project Team, Inria Centre Rennes-Bretagne Atlantique, Rennes, France; ^2^ SERPICO/STED Team, UMR144 CNRS Institut Curie, PSL Research University, Sorbonne Universites, Paris, France; ^3^ Inria, CNRS, IRISA, University Rennes, Rennes, France; ^4^ Laboratoire Physico-Chimie, Institut Curie, PSL Research University, Sorbonne Universites, CNRS UMR168, Paris, France; ^5^ CNRS, Inria, LISN, Université Paris-Saclay, Orsay, France; ^6^ LIRMM, Université Montpellier, CNRS, Montpellier, France; ^7^ MaIAGE, INRAE, Université Paris-Saclay, Jouy-en-Josas, France; ^8^ Decision and Bayesian Computation, Neuroscience and Computational Biology Departments, CNRS UMR 3571, Institut Pasteur, Université Paris Cité, Paris, France

**Keywords:** multi-dimensional biological data, virtual reality, augmented reality, intracellular imaging, bioimaging

## Abstract

Microscopy image observation is commonly performed on 2D screens, which limits human capacities to grasp volumetric, complex, and discrete biological dynamics. With the massive production of multidimensional images (3D + time, multi-channels) and derived images (e.g., restored images, segmentation maps, and object tracks), scientists need appropriate visualization and navigation methods to better apprehend the amount of information in their content. New modes of visualization have emerged, including virtual reality (VR)/augmented reality (AR) approaches which should allow more accurate analysis and exploration of large time series of volumetric images, such as those produced by the latest 3D + time fluorescence microscopy. They include integrated algorithms that allow researchers to interactively explore complex spatiotemporal objects at the scale of single cells or multicellular systems, almost in a real time manner. In practice, however, immersion of the user within 3D + time microscopy data represents both a paradigm shift in human-image interaction and an acculturation challenge, for the concerned community. To promote a broader adoption of these approaches by biologists, further dialogue is needed between the bioimaging community and the VR&AR developers.

## 1 Introduction

**FIGURE 1:**
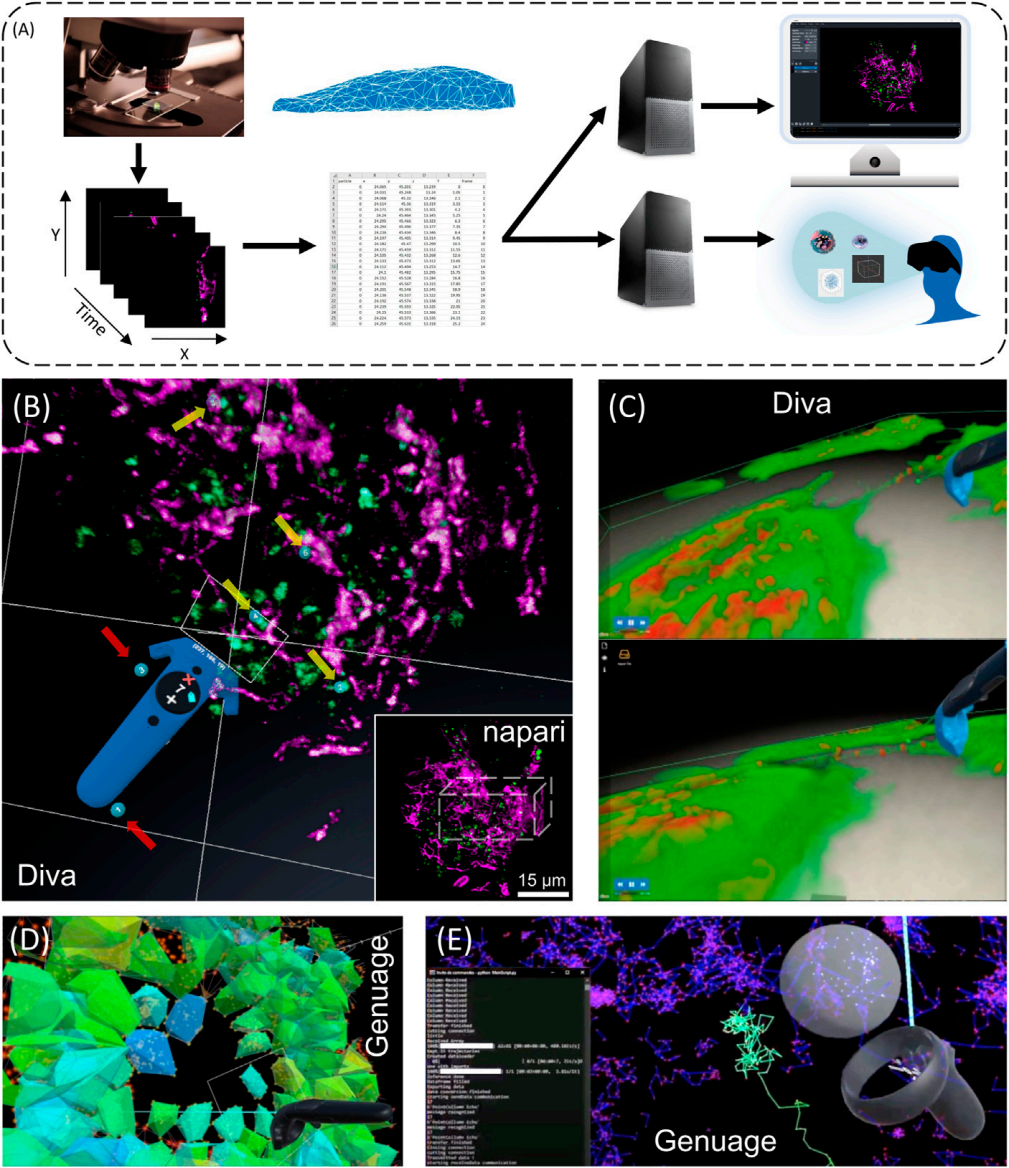
Exploring 3D + time intracellular microscopy data. **(A)** 3D + t visualization modes. Flat screen and VR/AR share identical processes. From left to right, image acquisition and preprocessing (denoising, registration, reconstruction, and deconvolution); post processing (segmentation, mesh, and tracking analysis) and data annotation constitute similar input. Fork in the road starts at a downstream computing level for 3D rendering, which differs between flatscreen and VR/AR visualizations, with additional interactive tools for the latter (e.g., Microsoft Hololens, Headsets, Oculus, eye trackers, haptic gloves) **(B)** VR visualization an interaction using Diva of a live RPE1 cell expressing CD63-Turquoise.2 labeling late-multivesicular endosomes and stained with PKMOrange (Liu et al., 2022), labeling mitochondria acquired with LLSM. Diving into the stack allows precise selection in 3D and it allows to adapt the angle of vision of green structures of interest (endosomes) (yellow arrows, numbered blue tags), accordingly to their distance to the magenta structures (mitochondria). Continuous visual penetration in the 3D space allows to precisely select more of them (yellow arrows, numbered blue tags) while previously selected ones remain tagged (red arrows, numbered blue tags). Coordinates and information of the selected structured are recorded for further analysis and tracking in time. In the inset thumbnail image, a corresponding full 3D stack of 54 planes of the same cell is shown using the napari viewer, where the parallelepiped white box depicts the volume manipulated in Diva. The same original 3D + t data set is also used in Figures 2D,E and in Supplementary Video S3. **(C)** Dynamic exchange of mitochondria between cells using Tunneling Nanotubes (TNT) (Courtesy of Chiara Zurzolo’s lab, Institut Pasteur) visualized with DIVA in VR (see Supplementary Video S1). Rendering is performed by full volumetric ray casting (el Beheiry et al., 2020) and an adapted transfer function (Guérinot et al., 2022). Note the possibility to have both the video running and the user interacting with the data. Exploring 3D single particle trajectories in time using Genuage **(D,E)**. Data are initially presented as point clouds and segment in Genuage, while physical properties maps **(D)**, such as coefficient diffusion are overlaid to the point cloud and explored in VR. **(E)** Example of real time analysis using a pre-trained neural network of a selection of points. Interaction with the data and rendering in VR is not affected during the analysis.

Imaging biomolecular dynamics in cells, such as intracellular trafficking, structural changes at the whole cell or intracellular level, has proven to be a challenge. A lack of sensitivity, limited recording speed, photobleaching and phototoxicity associated with conventional imaging have restrained, for a long time, our capacity to study biomolecules in their natural environments. Advanced approaches such as lattice light-sheet microscopes (LLSM), among others, overcome these difficulties while achieving a high spatial resolution (Chen et al., 2014). It is now possible to acquire 3D + time images for long periods (minutes to hours) at high frequency (milliseconds to seconds), opening new windows to understand fundamental mechanisms such as cell signaling, intracellular transport, and stochastic self-assembly in complex environments. It gives scientists the possibility to observe a series of sequential events, such as the complete vesicular transporter dynamics, from budding to final fusion within a whole cell, without space-time compromise. As another example, point cloud data, such as those generated by single-molecule localization microscopy (SMLM), are constantly increasing in size and dimension. Nevertheless, the current ways to interact and visualize these ever-growing multidimensional data sets are still limited and would deserve a more intuitive and interactive perception. Figure 1A shows a simplified comparative diagram of the integration paths for data visualization on flat screens and in VR/AR. The NAVISCOPE (“*Image guided NAvigation and Visualization data sets in live cell imaging and microscopy*”) project was initiated a few years ago to overcome the aforementioned visualization and interaction challenges. The consortium aims at developing visualization, navigation, and interaction methods to investigate temporal series of multi-valued volumetric microscopy images and facilitate their analysis. Several smart visualization methods have been developed over the years with the aim to gain easiness in navigation such as the VTK viewer (de Chaumont et al., 2012; Hanwell et al., 2015) or ClearVolume (Royer et al., 2015). Some commercial (Imaris, Aivia) or recently published (Leggio et al., 2019; Sofroniew et al., 2021), tools greatly improved navigation in imaging data sets (Supplementary Figure S1; same dataset visualized within ClearVolume, napari, Imaris and Aivia). With one exception, and limited to 3D visualization, ConfocalVR (Stefani et al., 2018), these approaches provide little or no support for VR/AR. Only a few years ago VR approaches such as scenery (Günther et al., 2019) and syGlass (Pidhorskyi et al., 2018) were applied to address the dynamics of cellular mechanisms. Other virtual reality developments were focused in SMLM (e.g., vLUME (Spark et al., 2020)). In Section 2, we review some of the visualization methods on flat screens and summarize their respective advantages but also limitations, with a focus on the temporal dimension. While not exhaustively, in Section 3, we then review a list of significant tools in VR, already published by co-authors of this perspective paper, Genuage (Blanc et al., 2020) and Diva (el Beheiry et al., 2020). In Section 4, we present augmented reality (AR) approaches, using MorphoNet and based on AR Foundation using the Unity3D Game engine or HoloTracks developed using Microsoft Hololens. Section 5 presents MorphoNetVR associated to a 3D timeline approach adapted to explore temporal data in VR (Fouché et al., 2022b).

Finally, we highlight the potential of the VR/AR approaches for scientists to 1) navigate into multidimensional, large data sets with another view angle or perception, 2) interact with these data especially by selecting subregions, 3) exploit these approaches to quantify features of interests, 4) validate data annotation and 5) detect artifacts not easily seen in flat screens. With this paper, we would like to share our belief that a close dialogue between bioimaging scientists and VR/AR specialists is now a hot topic in microscopy.

## 2 Exploring the temporal dimension in biological imaging on flat screens

Imaging technologies are producing various types of data including pixel-based images and point clouds localization data. Exploring and interacting with such multidimensional time-evolving datasets is a non-trivial task and retrieving useful information in real time faces many challenges. Commercial solutions (e.g., Imaris, Aivia) (Supplementary Figure S1C,D) deal with the visualization of large datasets including 3D time series, their rendering, analysis, statistical testing, and recently also AI/machine learning approaches. Other multidimensional image viewers are open-source libraries or software. ClearVolume (Royer et al., 2015) or napari (Sofroniew et al., 2021) are designed to browse, annotate, and analyze large multidimensional images (Supplementary Figure S1A,B). Because napari is tightly integrated with the *Python* ecosystem, innovative machine learning and analysis tools are easily coupled. The open-source web browser MorphoNet (Leggio et al., 2019) focuses on interactive visualization and sharing of morphodynamics datasets, onto which quantitative and qualitative information can be projected. Napari and MorphoNet are community-driven, the later exploiting already accessible or newly created natural or simulated morphological datasets. The exploration of the temporal dimension constitutes a difficult challenge (Andrienko et al., 2010). The usual methods of visualization and interaction, such as temporal sliders, animation, or the juxtaposition of a few time points, may be limited when analyzing dynamic features. Indeed, scrolling through time points but showing only the 3D data at one time point puts an important charge on short-term memory, yet typical display configurations limit the amount of time points that can be juxtaposed.

## 3 Immersive visualization of 3D + time data and analysis

The reason for using an immersive setup lies in the inherent benefits that an observer gains from a stereoscopic projection of 3D data (Dwyer et al., 2018). Recently, members of this consortium have introduced platforms generating volumetric reconstructions from stacks of 3D + t microscopic images and facilitating efficient visualization, selection of structures of interest and quantitative analysis [DIVA (el Beheiry et al., 2020)] (Figures 1B,C; Supplementary Video S1), as well as to visualize and analyze point clouds in VR, particularly adapted to SMLM data [Genuage (Blanc et al., 2020)] (Figures 1D,E). In both tools, data are imported in their raw format and visualized in VR using custom shaders. It goes beyond static data, as the visualization and interaction are optimized for dynamic data exploration over time (Figures 1C,E), combined with other information (Figures 1D,E). The challenge lies in memory management to maintain a comfortable VR experience with a refresh rate of 30–80 Hz while performing the various tasks. For point cloud representations, memory consumption can be evaluated in advance and the number of points to be displayed can then be adjusted. Other approaches to reduce the sampling of datasets can potentially reduce memory consumption but must be evaluated for proper visualization of data evolving over time. The possibility of VR to view the data stereoscopically and to interact within its volume can be essential to accelerate data annotation for machine learning pipeline. We recently showed that coupling data annotation in VR with one-shot learning (learning without a database) can significantly accelerate microscopy image segmentation. The process is being extended in systems where a limited database is accessible by joining VR and segmentation based on simplified UNet. Here is shown an application in time-evolving images (Figure 1E). Running the analysis in parallel to the VR visualization is challenging, as it impacts the fluidity of the rendering and the possibility to interact with the data. One approach relies on simulation-based inference to run analysis in space and time within the VR environment. Simulation-based inferences (Cranmer et al., 2020) rely on numerical simulations of the systems dynamics to train an inference procedure that can then be run in an amortized manner (usually by a neural network) when performing the inference (Blanc et al., 2022). This approach is instrumental when adding the time component in the analysis.

## 4 AR for immersive visualization of dynamic compounds in living cells

**FIGURE 2:**
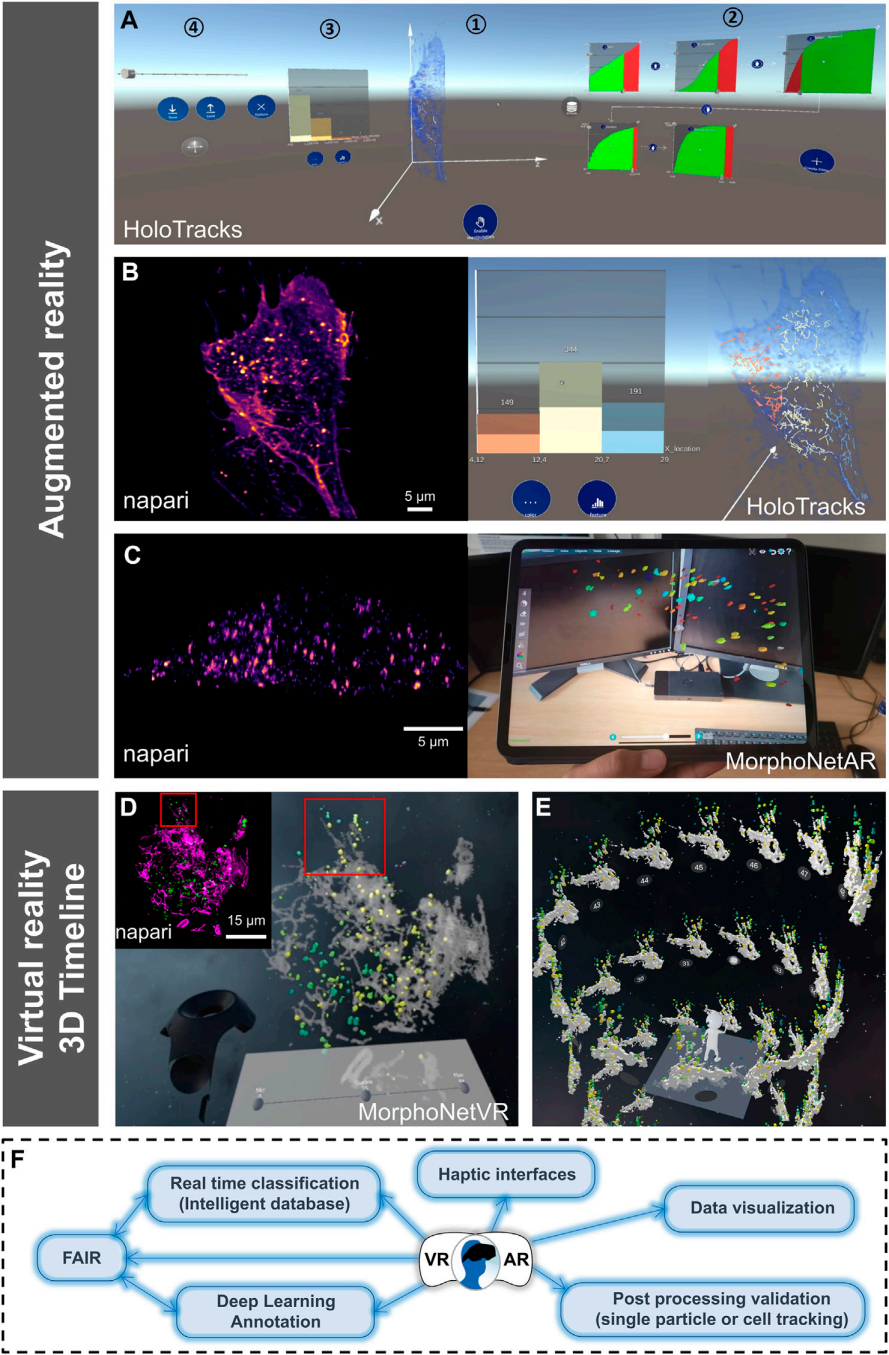
Other VR/AR developments and perspectives for VR/AR integration in the 3D + time digital microscopy landscape. **(A–C)** Augmented reality visualization (HoloTracks) of live RPE1 cell stacks acquired by Lattice Light Sheet microscopy after 1 h of incubation at 37°C with Plasma Membrane CellMask™ staining (ThermoFisher, Bordeaux, France) to label the endosomal pathway. Tracking of labeled vesicles was performed with Trackmate (Ershov et al., 2022). **(A)** Augmented Reality data dashboard that combines a 3D view (1) with several abstract data views (2 and 3) and interactive elements (1, 2, and 4) showing 3D view and possible data exploration (filter settings and resulting histograms) or view manipulations as facilitated by the Microsoft HoloLens 2 headset. **(B)** Paired images showing the same cell sample, using napari viewer (left image) and HoloTracks (right image) with color map of tracks visualization of moving endosomes and histograms of three distributions as selected by filtered settings. **(C)** Napari (left image) and augmented reality visualization using tablet technology (MorphoNetAR; right image) of one stack of a live Hela cell stably expressing eGFP-Rab5 acquired using LLSM. Data were segmented using llsmtools (Aguet et al., 2013) and the color map indicates volumes of Rab5 positive early endosomes (see Supplementary Video S2). **(D)** Quantitative visualization using virtual reality of a live RPE1 cell expressing CD63-Turquoise.2 labeling late-multivesicular endosomes and PKMOrange labeling mitochondria acquired with LLSM (MorphoNetVR). A full 3D stack of 54 planes at one time point is shown (see full movie in Supplementary Video S3). The color map of late endosomes represents the distance to mitochondria (green-far and yellow-near). Mitochondria network is represented in grey. The inset thumbnail image represents the flatscreen visualization using napari viewer of the same 3D stack with the same viewing angle (endosomes in green; mitochondria in magenta). **(E)** 3D Timeline design space in MorphoNetVR extracted from a subregion of interest indicated by boxes in **(D)** and in the thumbnail in **(D)**. **(F)** Schematic view of ongoing innovations and perspectives for VR/AR exploration of microscopy images.

As an approach to overcome some limitations of VR such as dizziness, isolation from the external environment, or computational constraints is the use of augmented reality (AR). An interface was designed (Figure 2A) for the interactive exploration of particle trajectories within living cells (e.g., endocytosis vesicles). Members of this consortium ultimately wish to evaluate collaborative data exploration scenarios (Sereno et al., 2022b) and thus used an AR headset, Microsoft’s HoloLens 2. This allows a semi-transparent volumetric representation of a cell that highlights its contour without losing the context of the surrounding room (Figures 2A,B; right image), to show the trajectories of the segmented and tracked objects of interest as opaque linear representations (Figure 2B; right image) while interacting with panning, zooming, and rotating. Finally, the temporal aspect of the data is explored using a slider. To create an immersive analysis environment, in addition to the 3D data display, it is also necessary to use abstract data visualizations (Dwyer et al., 2018). Specifically, a linked-view dashboard (Wills, 2008) was created that includes both 3D representations and abstract data views, with the latter serving primarily to provide context, filtering, and highlighting of data in the main 3D view (Figure 2A). To limit the set of trajectories displayed in the data and to focus on a particular subset, filtering modules were introduced. These modules consist of a visual representation of particular properties, such as average particle velocity, confinement rate, or displacement (Figure 2A, parts 2, 3). These properties were displayed for all trajectories as cumulative distribution functions. For each filter, a slider allows selection in the main 3D view by excluding trajectories below or above a threshold set for the corresponding property. Several of these filtering modules can be combined into a filtering pipeline. Of particular interest is the interaction design for the resulting data dashboard, which combines selection tools and 3D views into a single interface, but in an immersive space. Initially, all elements were arranged in a horizontal layout. However, as elements on the far left or right became difficult to reach, we curved the dashboard toward the observer (slightly curved in Figure 2A). Then, interactions that do not require much precision (e.g., pointing to a particular filter module) remain reachable, while items requiring fine-grained control are replicated in the center of the dashboard (Figure 2B; right images) during the interaction. For example, this replication was applied to the fine-tuning of filter modules (Figure 2A, part 2), which requires not only precise input but also observation of the module and of changes in the 3D view as well as quantitative histogram representation (Figure 2B; right image). AR can also exploit tablet technology (Sereno et al., 2022a) as illustrated here (Figure 2C; left image input data set as viewed in the napari viewer; right image, selected structures as a function of volume indicated by the color map, viewed on tablet using MorphoNetAR; Supplementary Video S2) using data launched in the web browser MorphoNet (Leggio et al., 2019).

## 5 From traditional to 3D timelines in VR for intra-cellular image data exploration

Like AR, VR is a powerful environment for designing complex visualization and interaction frameworks, capable of providing effective scientific analysis tools (Fonnet and Prié, 2021). In the NAVISCOPE consortium, we also proposed to extend timeline representations to temporal 3D microscopy data in VR. 2D Timelines typically display temporal information, e.g., a series of events, in a linear fashion along an axis or as a tree (Brehmer et al., 2017). However, current representations disregard the possibility to lay out the timeline such that they leverage the increased workspace provided by VR. In biological imaging, the design space must be revised to create visualizations representing the data as a series of time steps of the 3D temporal data along the governing curve of our timeline. It is thus needed to explore how to represent timelines in the virtual environment, including relying on different types of 3D curves, using different media to juxtapose multiple timelines, to exploit the large workspace of the virtual environment. The interaction capabilities of VR systems allow researchers to design interfaces for efficient exploration of 3D timelines (Fouché et al., 2022b). For example, users can easily move their point of view and look around, but also propose selection and manipulation techniques adapted to complex 3D data. This method is evaluated by creating an interactive visualization of a single-cell 3D temporal imaging LLSM dataset (up to 15 Gbytes), for two fluorescence channels, and corresponding to 1 volume per second over 2 minutes (Figures 2D,E). Structures (see the inset thumbnail presented in Figure 2D with the napari viewer) were segmented and the particles (endosomes) tracked with Trackmate (Ershov et al., 2022). Some information is calculated, such as the volume of structures in channel 1 (endosomes) or their distance to structures in channel 2 (mitochondria) (Figure 2D). This information can be encoded as a colormap. Using 3D selection techniques (Argelaguet and Andujar, 2013), users can select an area of interest at a specific time, for example by creating a bounding box (Figure 2D and inset in Figure 2D). The resulting area of interest, which evolves over time, constitutes the 3D data displayed at each time point in the timeline. Depending on the number of time points, one can choose a different shape for the timeline, here a helicoid taking advantage of the ascending dimension (Figure 2E). The timeline can be scrolled, by moving it along its guiding curve, and the content of each time point can be manipulated for instance by rotation (Supplementary Video S3). Filtering operations are also available to help the user refine the previously selected area of interest in space and time, by masking objects according to the color-mapped information to reduce the spatial dimensions, for instance. Overall, these operations and tools allow users to explore and manipulate 3D temporal data, define areas of interest, and analyze features (e.g., distances, movement) present in these complex datasets.

## 6 Discussion/perspectives

For VR/AR approaches to be adopted by the broader bioimaging community, it is important that they are evaluated by the biologists, on their own datasets. This is generally the case. However, while acknowledging the pioneering work done by interdisciplinary teams of artists and scientists (Cox, 1990) in the past, we argue here for the strengthening of continuous evaluation to co-construct more responsive VR/AR approaches that can address the ever-increasing size and resolution of 3D + time fluorescence microscopy data. For example, an extension of the well-known Space-Time Cube (STC) visualization technique has been developed for investigating cell divisions in morphogenesis analysis (Fouché et al., 2022a). Meanwhile, MorphoNetVR was adapted to intracellular dynamics together with the scientists who provided the datasets, and then tested by ten biologists at Institut Curie, working on different topics and who had never used these approaches before. The 3D timelines approach on regions of interest (Fouché et al., 2022b) was perceived as very efficient, especially by developmental biologists. Cell biologists brought remarks on the visualization of proximities and distances between different elements, which contributed to complete these visualizations by distance color-maps (Figure 2D). Immunologists insisted on the visualization of more than two elements at a time, as they often use fluorescence microscopy to look at up to 4 distinct cells and/or molecules. It is interesting that all of them asked for a return visualization of the “real data” by overlay/incrustation or on desktop, an experience shared for the development of DIVA, especially by medical doctors who wanted to directly relate the VR experience to their usual visualization procedure. We believe it is linked to acculturation that requires intensive training actions and precise tutorials but also closer collaborations between the communities. Yet, hybrid approaches combining AR/VR and 2D screen visualization might be a way for better adoption (Wang et al., 2020). Biologists also provided valuable feedback on “HoloTracks” (Figure 2A). In particular, while the stereoscopic view of the data is highly valued, the HoloLens’ default input mode of manual gestures in 3D space is not ideal for the intended application. Therefore, other forms of input should be explored, such as tablets (Sereno et al., 2022a) (Figure 2C, right image and Supplementary Video S2) or VR controllers in game-oriented headsets.

Why should cell biologists now be interested in these techniques? It is necessary to list their potential benefits for a field that has been very active in terms of technological advances, both in image acquisition and in innovative processing approaches, over the last 3 decades. The NAVISCOPE consortium aims to address this specific topic.

One of the very practical reasons why 3D + time VR/AR is now available to the bioimaging community is simply related to technological advances. Headsets are now relatively inexpensive (Stefani et al., 2018), software (Unity3D) and hardware (SteamVR) development tools are rapidly improving and code libraries are making the development of new applications easier and faster.

Furthermore, multidimensional image datasets from microscopy are becoming sufficiently annotated and integrated, increasingly accessible via intelligent databases (Williams et al., 2017), open to reuse [FAIR (Wilkinson et al., 2016)] and automated processing pipelines, needed upstream of transfer to VR/AR, steadily improved by an active community. Yet the whole picture is still dependent on imaging modalities, of which there are many. However, beyond the medical imaging communities, other communities close to cell biologists, such as structural biologists, have already incorporated VR/AR approaches into their research toolbox (Zhang et al., 2019; Cortés Rodríguez et al., 2022).

Among advantages of VR/AR, the dynamics of cellular processes can be observed from all angles, in an immersive context, in different “perspectives”, spherical, convex or concave, and brings the peripheral vision. This facilitates much more human-data interaction as compared to flat screen navigations. New VR/AR approaches (Genuage and HoloTracks) also provide specific quantification tools to show distances, angles, counting, local density, and histogram profiler or include a selection of regions of interest for further analysis such as the 3D Timelines (Fouché et al., 2022b). Because communication with analysis software coded in Java or *Python* is now integrated, more post-treatment analysis is possible on selected features.

We can foresee ongoing and future innovation avenues:

A) There are multiple ways to improve microscopy images rendering in VR/AR, such as better integration of multiple channels (more than 2) with high pixel resolution (>16 bits/channel) or the addition of vector representations, which could add information about the orientation, movement of molecules or organization of structures such as cytoskeleton elements or membrane lipids [e.g., polarized microscopy, electric fields (EFs)]. Some authors have already started to explore this path using vector representations (Blanc et al., 2020).
B) There is potential to improve the interaction between humans and VR/AR, using eye-tracking (Günther et al., 2020) or through haptic interfaces (Petit et al., 2020). For example, in more biophysical applications, while measuring forces in cells or multicellular systems using optical tweezers or microchips is widely used, kinesthetic communication could bring human perception by providing local sensations, which would further improve the selection of responses in a 3D + time space.
C) VR offers visualization and interaction capabilities that should facilitate data annotation by providing better classification validation, in terms of morphological, tracking, and vector features, among others. The combination of this additional information would improve training datasets for deep learning approaches, if, however, the size of the datasets that can be visualized and interacted within VR is increased.
D) Finally, if considering VR/AR approaches as an interesting way to improve microscopy data annotation, feedback information must be linked to the input dataset, meaning refreshing a database connected to the VR system, via Cloud technology (Guérinot et al., 2022). This could be potentially achieved close to real time, through a hybrid technology, combining VR/AR and visualization on 2D screens. Then, the traceability process along the full life cycle of image data will include VR/AR output.

## Data Availability

The datasets presented in this study can be found in online repositories. The names of the repository/repositories and accession number(s) can be found below: The datasets used for this study can be found in the figshare repository with the identifier doi: 10.6084/m9.figshare.20290155. Rab5 endosomes data can be accessed through MorphoNet webserver [https://morphonet.org/].
