# Supplementary material for: Challenges of intracellular visualization using virtual and augmented reality
Source: Front Bioinform. 2022 Sep 13;2:997082. doi: 10.3389/fbinf.2022.997082 (PMC9580941; doi:10.3389/fbinf.2022.997082)
Supplement: Supplementary file 2 [file DataSheet1.PDF]

Supplementary Material

1 Introduction

This supplementary material shows examples of significant tools for 3D visualization, both flat screen and VR/AR approaches, presented in the main manuscript. We focused on intracellular trafficking.

2 Supplementary Figure

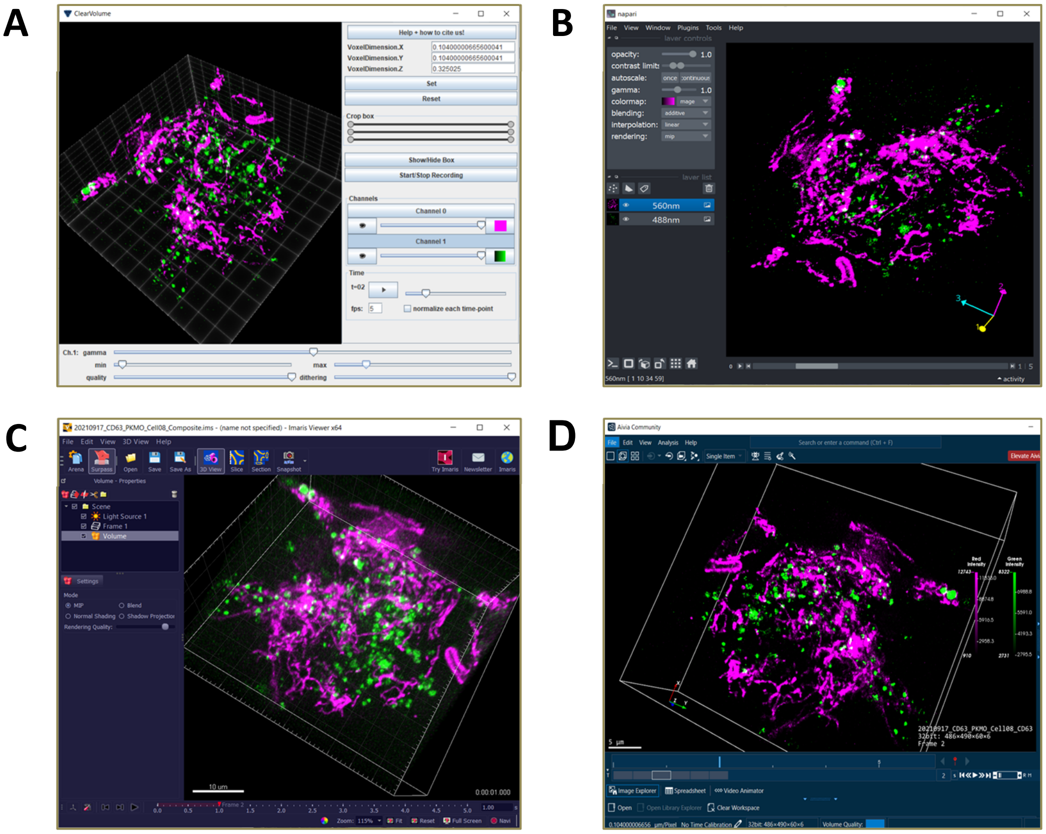

Supplementary Figure 1. Examples of widely used desktop visualization tools showing one full stack (54 planes) of live RPE1 cell stained with CD63-Turquoise.2 (green) for endosomes and PKMOrange (Liu et al., 2022) (magenta) for mitochondria (image data provided by the authors), acquired with Lattice Light-Sheet (LLS) microscopy. This stack corresponds to one time point extracted from 100 in the data set. (A) ClearVolume (Royer et al., 2015) and (B) napari (Sofroniew et al., 2021), both "open-source" software platform, (C) Imaris and (D) Aivia, as commercial solutions.

3 Supplementary Videos

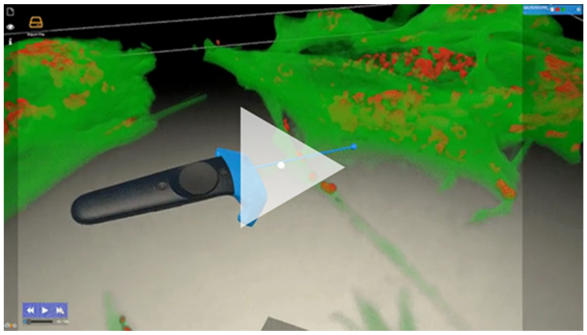

Supplementary Video 1. Video of dynamic exchange of mitochondria between cells using Tunneling Nanotubes (TNT) (Courtesy of Chiara Zurzolo's lab, Institut Pasteur) visualized with DIVA (el Beheiry et al., 2020) in virtual reality.

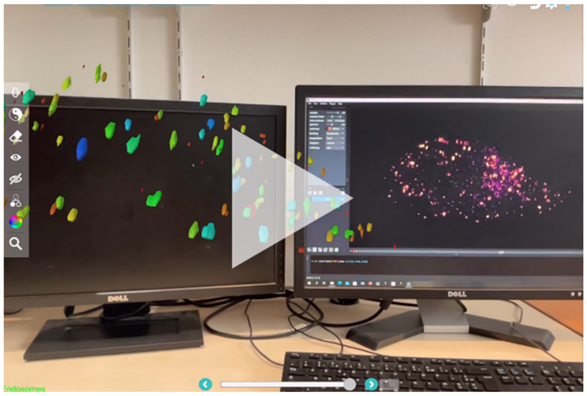

Supplementary Video 2. Augmented reality visualization (MorphoNetAR) of a live Hela cell stably expressing eGFP-Rab5 acquired using LLSM (full original data set of 50 stacks each constituted of 56 planes) paired with a flat screen visualization using napari viewer, at the back. Color map indicates volumes of Rab5 positive early endosomes.

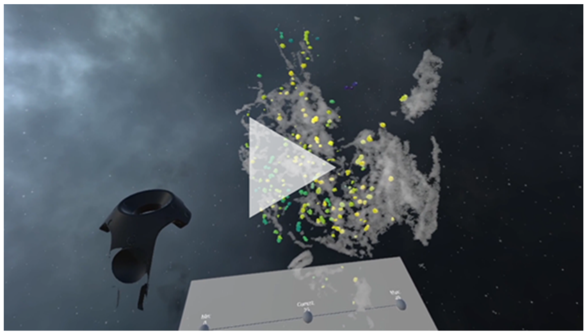

Supplementary Video 3. Quantitative visualization using virtual reality of a live RPE1 cell expressing CD63-Turquoise.2 labeling late-multivesicular endosomes (green) and PKMOrange (gray) labeling mitochondria acquired with LLSM (full original data set of 100 stacks each constituted of 54 planes) using MorphoNetVR. Late endosome color map represents distance to mitochondria (green-far and yellow-close). In the second part of the video, timelines of a region of interest from the same series are indicated.

4 References

el Beheiry, M., Godard, C., Caporal, C., Marcon, V., Ostertag, C., Sliti, O., et al. (2020). DIVA: Natural Navigation Inside 3D Images Using Virtual Reality. *Journal of Molecular Biology* 432, 4745–4749. doi: 10.1016/j.jmb.2020.05.026.

Liu, T., Stephan, T., Chen, P., Chen, J., Riedel, D., Yang, Z., et al. (2022). Multi-color live-cell STED nanoscopy of mitochondria with a gentle inner membrane stain. *bioRxiv*, 2022.05.09.491019. doi: 10.1101/2022.05.09.491019.

Royer, L. A., Weigert, M., Günther, U., Maghelli, N., Jug, F., Sbalzarini, I. F., et al. (2015). ClearVolume: open-source live 3D visualization for light-sheet microscopy. *Nature Methods* 12, 480–481. doi: 10.1038/nmeth.3372.

Sofroniew, N., Lambert, T., Evans, K., Nunez-Iglesias, J., Bokota, G., Peña-Castellanos, G., et al. (2021). napari/napari: 0.4.12rc2. doi: 10.5281/ZENODO.5587893.
